# Supplementary material for: Effects of Eucalyptus Biochar on Intestinal Health and Function in Largemouth Bass (Micropterus salmoides)
Source: Biology (Basel). 2025 Dec 7;14(12):1754. doi: 10.3390/biology14121754 (PMC12731124; doi:10.3390/biology14121754)
Supplement: Supplementary file 1 [file biology-14-01754-s001.zip › biology-3976500-supplementary.pdf]

Table S1. Physical and chemical properties of biochar.

Items	Numerical Value
pH	7.87
Moisture (%)	5.82
Ash (%)	13.28
Total organic carbon (%)	40.33
Total nitrogen (%)	0.845
Total phosphorus (%)	0.413
Total potassium (g kg^{-1})	27.31
Cu (g kg^{-1})	0.11
Mn (g kg^{-1})	0.14
Pb (mg kg^{-1})	6.09
Fe (mg kg^{-1})	5.62
Specific surface area (m^2 g^{-1})	20.78
